# Osmophobia in primary headache patients: associated symptoms and response to preventive treatments

**DOI:** 10.1186/s10194-021-01327-2

**Published:** 2021-09-18

**Authors:** Marianna Delussi, Anna Laporta, Ilaria Fraccalvieri, Marina de Tommaso

**Affiliations:** Applied Neurophysiology and Pain Unit, SMBNOS Department, Aldo Moro University, Bari, Italy

**Keywords:** Migraine, Osmophobia, Observational study, Preventive treatment

## Abstract

**Background:**

Osmophobia, is common among primary headaches, with prevalence of migraine.

The study aimed to evaluate prevalence and clinical characteristics of patients with osmophobia in a cohort of primary headache patients selected at a tertiary headache center. The second aim was to verify the possible predicting role of osmophobia in preventive treatment response in a sub cohort of migraine patients.

**Methods:**

This was an observational retrospective cohort study based on data collected in a tertiary headache center.

We selected patients aged 18–65 years, diagnosed as migraine without aura (MO), migraine with aura (MA) or Chronic Migraine (CM), Tension-Type Headache (TTH); and Cluster Headache (CH). We also selected a sub-cohort of migraine patients who were prescribed preventive treatment, according to Italian Guidelines, visited after 3 months follow up.

Patients were considered osmophobic, if reported this symptom in at least the 20% of headache episodes. Other considered variables were: headache frequeny, the migraine disability assessment (MIDAS), Allodynia Symptom Checklist, Self-rating Depression scale, Self-rating Anxiety scale, Pain intensity evaluated by Numerical Rating Scale-NRS- form 0 to 10.

**Results:**

The 37,9% of patients reported osmophobia (444 patients with osmophobia, 726 without osmophobia).

Osmophobia prevailed in patients with the different migraine subtypes, and was absent in patients with episodic tension type headache and cluster headache (chi square 68.7 DF 7 *p* < 0.0001). Headache patients with osmophobia, presented with longer hedache duration (F 4.91 p 0.027; more severe anxiety (F 7.56 0.007), depression (F 5.3 p 0.019), allodynia (F 6 p 0.014), headache intensity (F 8.67 p 0.003). Tension type headache patients with osmophobia (n° 21), presented with more frequent headache and anxiety. A total of 711 migraine patients was visited after 3 months treatment. The change of main migraine features was similar between patients with and without osmophobia.

**Conclusions:**

While the present study confirmed prevalence of osmophobia in migraine patients, it also indicated its presence among chronic tension type headache cases, marking those with chronic headache and anxiety.

Osmophobia was associated to symptoms of central sensitization, as allodynia. It was not relevant to predict migraine evolution after first line preventive approach.

**Supplementary Information:**

The online version contains supplementary material available at 10.1186/s10194-021-01327-2.

## Background

Osmophobia, defined as a fear, aversion, or psychological hypersensitivity to odors, is a very rare isolated phobia. It is common among primary headache patients, with prevalence of migraine. Its inclusion among diagnostic criteria was suggested, based on evidence of specificity for migraine diagnosis, greater than photophobia and phonophobia [1, 2]. In fact, the isolated presence of osmophobia during headache attacks should be considered a diagnostic criterion [2].

Recent literature showed an association between osmophobia and symptoms of central sensitization, as allodynia [2]. Other associated features, as higher pain intensity and frequency of migraine, indicated osmophobia as a possible marker of severe migraine [3–5]. In general, osmophobic patients have a more florid clinical picture and more affective symptoms [6]. In fact, migraine patients with osmophobia were more likely to have higher levels of depression and anxiety than those without osmophobia [7]. Osmophobia may even have a prognostic role during migraine chronicization [3].

First line preventive treatment for migraine includes beta blockers, calcium channel blockers, antiepileptics and antidepressants, according to current national guidelines [8]. In a recent observational retrospective study, we observed a mild improvement of migraine frequency and disability after 3 months follow up, while the presence of allodynia predicted a poorer clinical outcome.

Osmophobia was associated to severe migraine and allodynia, so its presence could contribute to early detect potential therapeutic failure and drug resistant patients, also in view of the use of new available therapies [9].

### Aim of the study

The first aim of this study was to further evaluate prevalence and clinical characteristics of patients with osmophobia in a cohort of primary headache patients selected at a tertiary headache center. The second aim was to verify the possible predicting role of osmophobia in preventive treatment response in a sub cohort of migraine patients. We hypothesized that osmophobia, other than frequent among migraneurs, could be a marker of disease severity and possible drug resistance.

## Method

### Study design

This was an observational retrospective cohort study based on data collected in a tertiary headache center, Applied Neurophysiology and Pain Unit (ANPlab), Policlinico General Hospital of Bari.

The local Ethics Committee of Bari Policlinico General Hospital approved the use of the electronic database, and patients signed an informed consent form about the inclusion of their data and use for scientific purposes.

### Study population

The present data were extracted from an electronic data set collected from September 2017 to October 2020. The clinical features were converted into electronic codes useful for retrospective analysis. For the present analysis, we selected patients aged 18–65 years, who came for the first time to the Bari Policlinico General Hospital and who received a diagnosis of: migraine without aura (MO), migraine with aura (MA) or Chronic Migraine (CM), Tension-Type Headache (TTH- Episodic Tension Type Headache-ETTH; Chronic Tension Type Headache - ETTH), Cluster Headache (CH) and other forms of primary headache [9, 10]. We did not select patients with severe general medical diseases, such as hepatic, renal and cardiovascular insufficiency; previous or current neurologic diseases beside migraine; a diagnosis of current or previous psychiatric diseases; any disease with potential olfactory failure, which we specifically checked for among the comorbidities reported in the electronic database.

We also selected a sub-cohort of migraine patients (migraine with aura, without aura and chronic migraine, according to current International Classification [11], who were prescribed preventive treatment, according to Italian Guidelines [8]. They were out patients reporting more that 4 migraine attacks in the 3 months preceding the first visit, without history of previous treatments. They were visited after 3 months follow up. Some of the patients were also included in the previous evaluation, and we followed the same therapeutic options detailed in Delussi and de Tommaso [12]. Patients were considered responders if they reported a 50% reduction of headache frequency at follow up.

### Clinical assessment

Patients underwent the clinical assessment that we described in previous studies [12]. At the moment of visit booking, patients are generally requested to fill a headache diary [12–14]. The same chart, is recommended to be completed during the follow up period. The diary includes the allodynia scale with scores from 0 to 12, according to previous studies [14, 15], and presence of symptoms associated with headache, as osmophobia, for single headache episodes. Clinical features are checked from the diaries, but patients are interviewed again, as in many cases, they are not confident or compliant with data collection. Neurologists with clinical experience in headache, put the diagnosis of headache based on characteristics and frequency, according with the International Headache Society criteria [10, 11] and complete the electronic sheet. Patients were considered osmophobic, if reported this symptom in at least the 20% of headache episodes [1]. The considered variables were: The migraine disability assessment (MIDAS) [16], Allodynia Symptom Checklist, [17], Self-rating Depression scale [18], Self-rating Anxiety scale [19], Pain intensity evaluated by Numerical Rating Scale-NRS- form 0 to 10.

### Statistic analysis

Demographic and basal clinical data of patients included in the different headache groups, were evaluated by one way ANOVA with a post hoc Bonferroni test.

We used the chi square test to assess the presence of osmophobia among different headache subtypes. The MANOVA analysis estimated the clinical and demographic variables as age, headache duration, headache frequency, allodynia, SAS, SDS, MIDAS, NRS, taking into consideration the presence of osmophobia and headache diagnosis as factors.

A MANOVA analysis determined also the effect of osmophobia on mean clinical features in migraine patients after 3 months preventive therapy follow up, considering also single drugs as a factor. In MANOVA, the Pillais trace was considered.

A discriminant analysis with leave one out method and Wilks lambda served to identify basal migraine features, including osmophobia, characterizing responder patients at 3 months follow up.

## Results

### Demographic data for selected patients

**Fig. 1:**
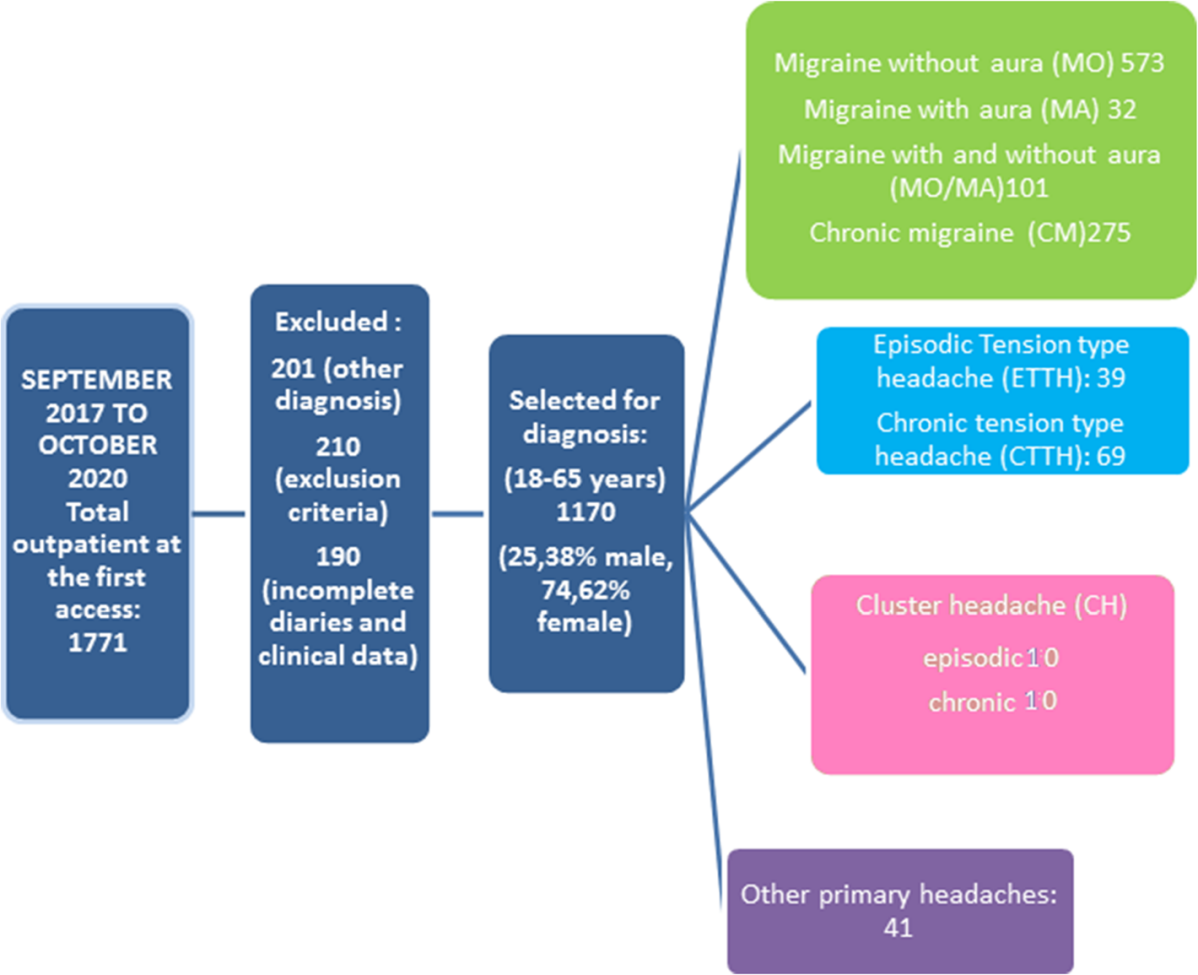
Flow chart: patients’ selection

The flowchart depicting patient selection is shown in Fig. 1.

Among CH patients, 10 were episodic and 10 were chronic. All patients were visited during active cluster. Among other headaches, 15 patients had primary stabbing headache, 2 patients had hypnic headache, 7 patients had primary thunderclap headache, 6 patients were diagnosed as chronic paroxysmal hemicrania, 7 patients as hemicranias continua, 2 patients had primary cough headache, 2 patients primary exertional headache.

### Osmophobia in headache patient cohorts

The 37,9% of patients reported osmophobia (444 patients with osmophobia, 726 without osmophobia). Osmophobia prevailed in females (28.2% males, 42.7% females, chi square 19.9 *p* < 0.001).

Osmophobia prevailed in females (28.2% males, 42.7% females, chi square 19.9 *p* < 0.001).

**Fig. 2:**
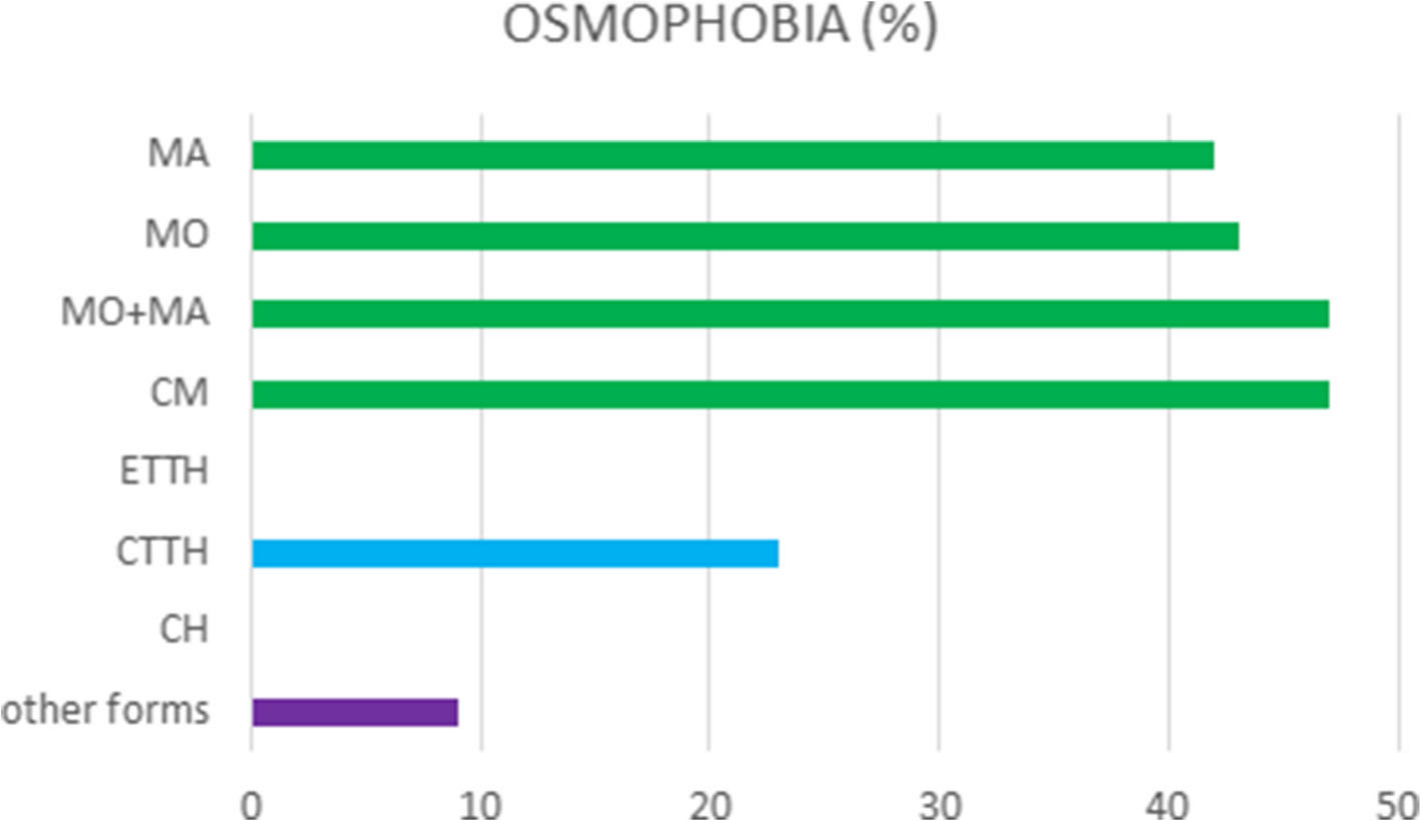
Prevalence of osmophobia among different diagnosis. MO Migraine WithOut Aura. MA Migraine With Aura. CM: Chronic Migraine. ETTH: Episodic Tension Type Headache. CTTH: Chronic Tension Type Headache. CH: Cluster Headache

The chi square test showed that osmophobia prevailed in patients with the different migraine subtypes, and was absent in patients with episodic tension type headache (ETTH) and cluster headache (CH) (chi square 68.7 DF 7 *p* < 0.0001) (Fig. 2). Among the other forms, the 4 osmophobic patients had a diagnosis of primary stabbing headache.

### Clinical characteristics of osmophobic patients

The MANOVA analysis, showed that the considered variables were different among headache groups and among patients presenting or not with osmophobia.

(Diagnosis as factor F –Pillais- 6.74 DF 91 *p* < 0.001; osmophobia as factor F 2.68 DF 13 *p* < 0.0001).

Clinical variables were different among headache groups (Tables 1S and 2S). In particular patients with CM confirmed more severe disability, allodynia and pericranial tendency.

**Fig. 3:**
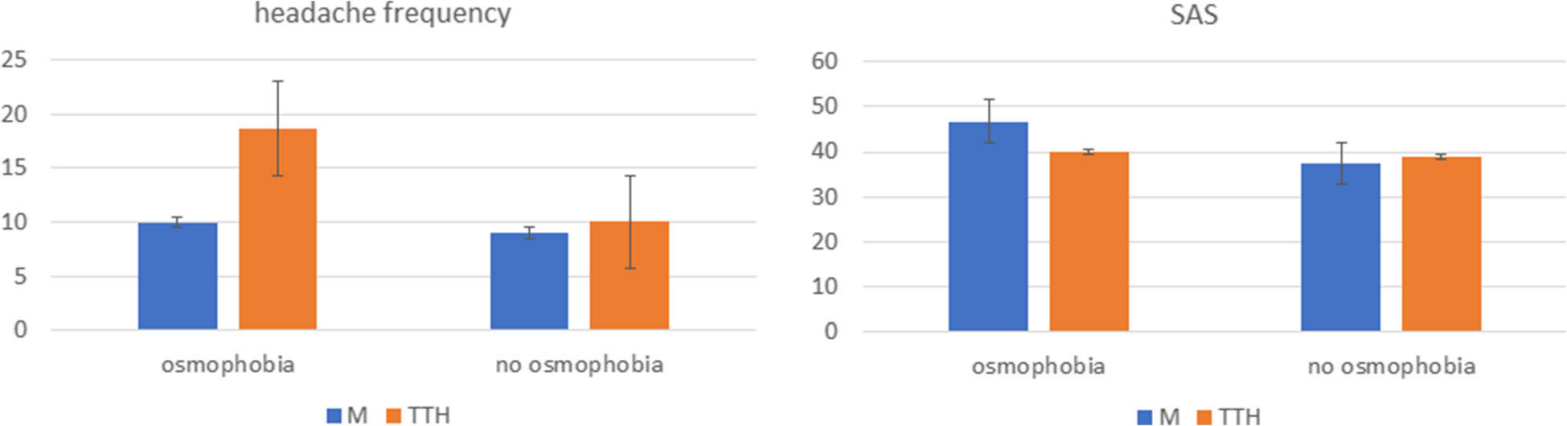
Mean and Standard deviations of headache frequency and anxiety disorder in patients with osmophobia e without osmophobia

The interaction diagnosis x osmophobia was not significant when single groups were considered. (F 1.16 DF 65 p 0.17), Merging headache subgroups into main diagnosis (Migraine, Tension type headache, cluster headache), the interaction diagnosis x osmophobia was significant (F 18.5 p 0.016). Headache patients with osmophobia, presented with longer hedache duration (F 4.91 p 0.027; more severe anxiety (F 7.56 0.007), depression (F 5.3 p 0.019), allodynia (F 6 p 0.014), headache intensity (F 8.67 p 0.003). Tension type headache patients with osmophobia (n° 21), presented with more frequent headache and anxiety as compared to those without osmophobia (Fig. 3).

In the migraine group, including MO, MA, CM and MO/MA, patients with osmophobia were older, with longer migraine history, more severe disability, anxiety, depression and allodynia (Table 1).

**Table 1:** Mean, Standard Errors, 95% Confidence Intervals and results of ANOVA analysis for single variables in the total cohort of migraine patients, including MO, MA and CM

osmophobia		mean	ds	95% CI		ANOVA	p
				lowe	higher		
age	YES	40.6	0.7	39.2	42.03	7.84	0.01
	NO	37.9	.0.6	36.74	39.23		
duration	YES	19.29	0.71	17.89	20.68	45.06	< 0.001
	NO	12.87	0.64	11.62	14.13		
frequency	YES	9.93	0.49	8.96	10.90	1.70	0.19
	NO	9.07	0.44	8.20	9.94		
MIDAS	YES	28.11	1.84	24.49	31.73	4.97	0.03
	NO	22.59	1.66	19.34	25.84		
SAS	YES	39.83	0.45	38.96	40.71	4.43	0.04
	NO	38.57	0.40	37.78	39.36		
SDS	YES	42.21	0.42	41.38	43.04	8.88	< 0.001
	NO	40.52	0.38	39.77	41.26		
allodynia	YES	4.05	0.14	3.78	4.31	38.34	< 0.001
	NO	2.92	0.12	2.68	3.16		
Pain intensity	YES	8.87	0.09	8.68	9.05	17.41	< 0.001
	NO	8.34	0.08	8.17	8.50		

### Predictive role of osmophobia on migraine outcome after 3 months preventive treatment

**Fig. 4:**
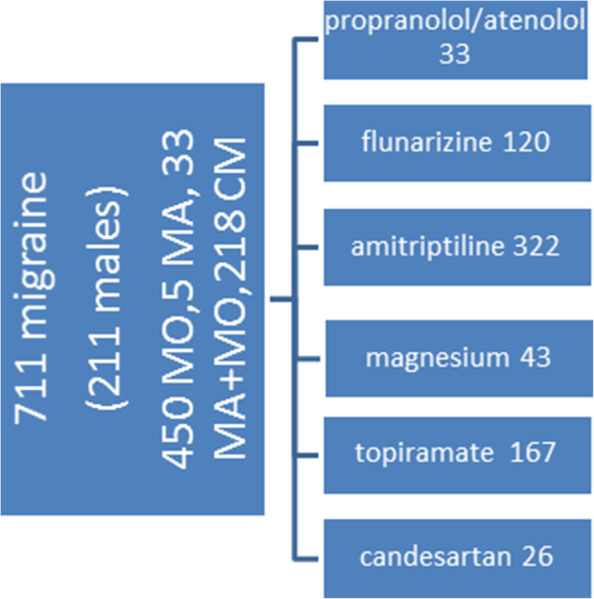
Patients and preventive therapy stratification

A total of 711 migraine patients was visited after 3 months treatment (Fig. 4).

Single migraine features and used drugs are reported in supplementary Tables. There was a global improvement of main migraine variables, with a prevalent effect of amitriptyline on depression, and a better effect of flunarizine in respect to other treatments on headache frequency (Tables 3S and 4S).

The most of patients with osmophobia at baseline, presented it at follow up (87.7%). The few patients reverted to not osmophobic, were equally distributed among the different treatment groups (chi square 7.15 p 0.2).

**Fig. 5:**
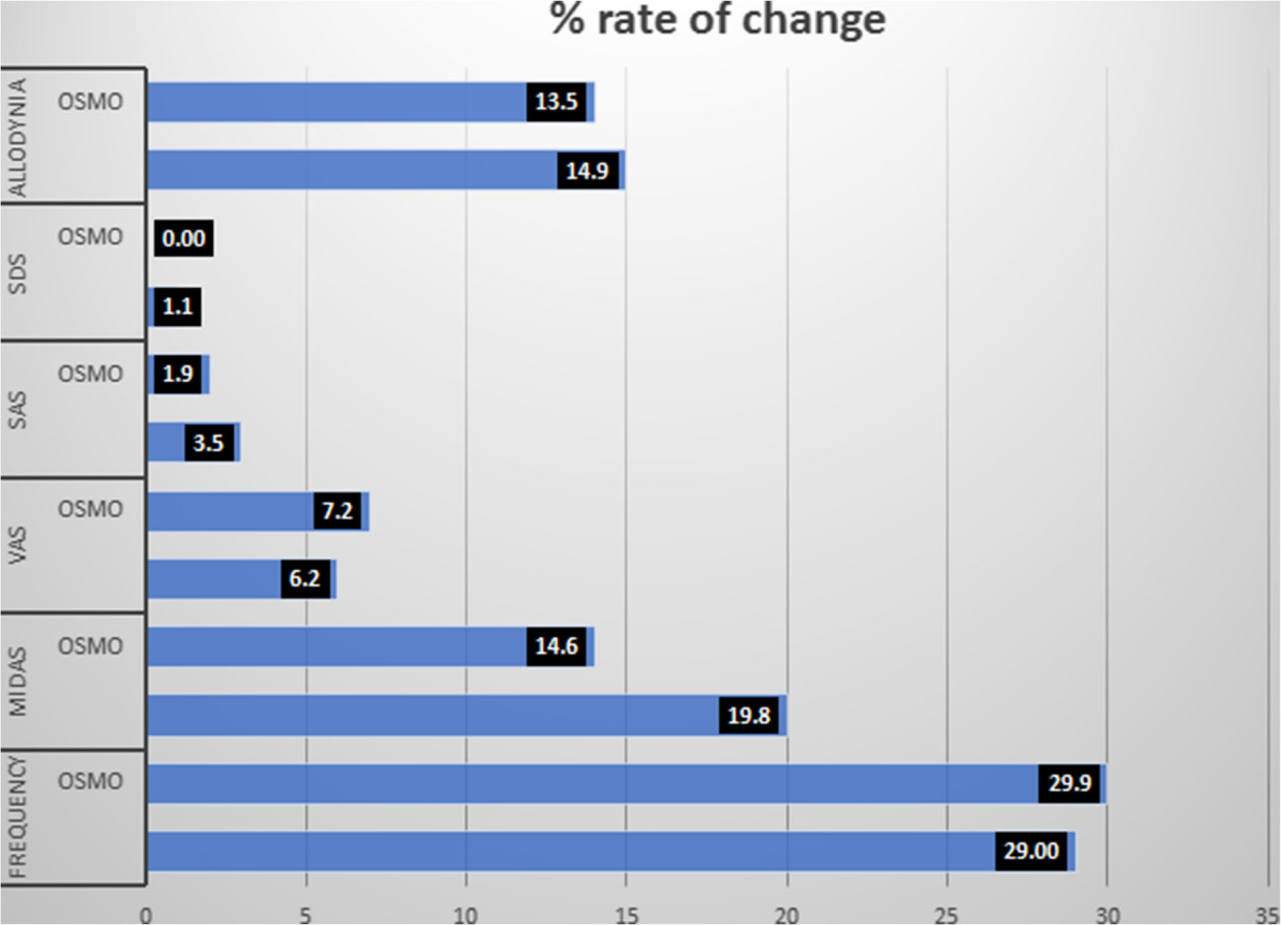
Response to preventive treatments in osmophobic and non-osmophobic subjects, computed as the percent rate of monthly headache frequency reduction

The MANOVA analysis with osmophobia and preventive drugs as factors, showed that the change of main migraine features- migraine frequency, VAS, MIDAS, allodynia, anxiety and depression- after 3 months preventive treatment, was similar between patients with and without osmophobia (MANOVA with osmophobia as factor: F 1.59 p 0.14; treatment as factor: F 1.61 p 0.019) (Fig. 5).

The interaction osmophobia x preventive treatment was also not significant (F 0.92 p 0.59).

Patients with 50% of headache frequency improvement were 250 (35,2%).

Patients with osmophobia had a not relevant risk to be non responder to first preventive aid (odds ratio 1.09).

Higher allodynia scores at baseline (Wilks Lambda - F 18.42 *p* < 0.0001), correctly classified the 67,3% of migraine patients with < 50% of frequency reduction. Osmophobia, frequency of headache, anxiety, depression, pain rating and MIDAS at baseline were excluded for the low discriminant performance.

## Discussion

Results of present observational study in a cohort of primary headache patients, confirmed what described in previous studies, an association between osmophobia and headache duration and intensity and allodynia, with a prevalence of osmophobia in migraine patients. Among tension type headache patients, osmophobia characterized patients with chronic form and higher levels of anxiety.

In our migraine gropu, osmophobia did not represent a risk for preventive treatment failure.

The discussion is detailed below.

### Osmophobia in headache cohorts

In line with previous works [1–6, 20–29] our data show a prevalence of osmophobia in migraine patients and differences in clinical features of osmophobic headache patients in respect to non-osmophobic ones.

No cluster headache patients, or patients included in “other forms” group and diagnosed as trigeminal autonomic cephalgia reported osmophobia. Present data confirm that osmophobia is not a symptom of trigeminal autonomic cephalgia [7, 30], rather in rare cases its presence could suggest a mixed form [31]. Among other forms, 4 cases with primary stabbing headache reported osmophobia. This is an infrequent primary headache, which can be associated with migraine. While the presence of osmophobia alone did not presently support the diagnosis of migraine [2], it could suggest the possibility that migraine attack could in perspective occur [32].

### Clinical characteristics of osmophobic patients

Presence of osmophobia, was associated with older age, pain intensity, length of headache history, disability,anxiety and depression symptom, allodynia. Considering the large prevalence of osmophobia among migraine patients, its presence indicated a more severe migraine, in terms of single attack intensity and disability, more than in terms of frequency. Episodic and chronic migraine had a similar representation of osmophobia, according to previous studies [33]. These authors, found an inverse relationship between the presence of accompanying symptoms and frequency of migraine, as in the transition from episodic to chronic migraine, single attack intensity and severity tends to diminish, while their number increases. An inverse association between chronic form and osmophobia was found in tension type headache group. While few patients with tension type headache were osmophobic, all of them were chronic. Considering that the most of studies defined osmophobia as a specific clinical marker of migraine [1, 2], those chronic tension type headache patients with referred osmophobia should be reconsidered in view of associated migraine. In our clinical practice, we take particular attention to symptoms of migraine, all reported in our electronic medical record. However, current diagnostic criteria do not consider the isolated presence of osmophobia as conclusive for migraine [2, 11]. Osmophobic tension type headache, were also more anxious than non osmophobic ones, a factor predisposing to central sensitization signs and chronic evolution [34]. As a matter of fact, the link beetween osmophobia and signs of central sensitization as allodynia was very strong in the whole of headache patients and particulary in migraine [6, 7, 35]. Allodynia is the most evident clinical manifestation of central sensitization that in turn is a process of progressive dysfunction in pain processing modality, at least in predisposed subjects [4].

Osmophobic patients seem to have a more florid clinical picture and more affective symptoms. These findings suggest that Osmophobia is related to a broader sensorial hypersensivity which include photophobia, phonophobia, and allodynia during migraine attacks [36–38] evolving in the course of the disease [5, 39].

As a rule, migraineurs have a dysfunctional cortical processing in response to stimulation with various sensory modalities during attacks as well as interictally [40].

The significant association of osmophobia with affective symptoms is also worthy of comments.

Functional imaging studies during migraine attacks have revealed activation in areas such as the insula, thalamus, cingulate cortex [41–43]. In mood and anxiety disorders, several limbic areas have been found altered (e.g. amygdale, anterior cinguli, periacqueductal gray) [41]. Smell is innate related to limbic system [41]. Olfactory hypersensitivity, anxiety and pain share common neural pathways and area activation, and a possible functional association and interaction one each other might be argued.

#### Osmophobia as predictive factor for preventive treatment effect

In regard to the migraine cohort evaluated after 3 months preventive treatment, it is not a primary aim of the study to give comments about the effect of therapies. Briefly, present study confirmed what recently reported by our group, a high percentage of patients below the threshold of 50% headache frequency reduction, though in the present evaluation we found more responders [12]. There was also a general mild improvement of main migraine clinical aspects, while flunarizine confirmed a better effect on frequency and amitriptyline on mood.

At the best of our knowledge, it is the first study evaluating the role of osmophobia in predicting the response to preventive treatments. Our results exclude that its presence may be associated with the low treatment effect. Osmophobia marked more severe migraine in baseline condition, but its presence is unrelevant for drugs response at the follow up. Allodynia was the most reliable predictor of treatment failure. Allodynia remains a robust indicator of central sensitization and symptomatic and preventive treatment efficacy [12, 44, 45]. The other potential facilitating and associated factors, as anxiety, depression, or hypersensitivity symptoms as osmophobia, are features of severe migraine, but they are not predictive factors for treatment failure. The most of patients followed our indication of the use of triptans. The effect of symptomatic drugs resulted from the average intensity of attacks, which had a mild improvement. Osmophobia tended to persist in the most the attacks, in spite of headache intensity reduction. Moreover, considering the osmophobic and not osmophobic, patients had quite the same clinical outcome; its persistence could be considered not relevant for the global effect of treatments.

### Study limitations

Diaries are often difficult to be completed by patients, and they frequently did not report all attacks and symptoms during single headache episodes. Moreover, in their first access, neurologists check for diaries accuracy and interview patients again. In case of osmophobia, usually they ask patients to confirm its presence in the majority of critical episodes. Headache groups were very different in numbers, and migraine patients prevailed, as is usual in tertiary headache centres, so data of osmophobia prevalence need to be confirmed in studies conducted among general population.

## Conclusions

While the present study confirmed prevalence of osmophobia in migraine patients, it also indicated its presence among chronic tension type headache cases, marking those with chronic headache and anxiety. These patients could be prospectively observed for the possible presence of associated migraine or poorer clinical evolution.

Osmophobia shows strict relationships with symptoms of central sensitization, as allodynia, and factors facilitating it, as psychopathological traits, but it is equally represented among episodic and chronic migraneurs.

It is not relevant to predict migraine evolution after first line preventive approach. Even though osmophobia has a strict relation with allodynia, the latter alone remains the most robust predictive feature of drugs failure.

## Supplementary Information

**Additional file 1: Table 1S.** Clinical variables in the headache groups. **Table 2S.** Clinical variables in the headache groups. **Table 3S.** Results of repeated measures ANOVA evaluating main effect of preventive drugs on migraine features. **Table 4S.** Results of repeated measures ANOVA evaluating main effect of preventive drugs on migraine features.

## Data Availability

Data are available on request.

## Abbreviations

MO: Migraine WithOut Aura
MA: Migraine With Aura
CM: Chronic Migraine
ETTH: Episodic Tension Type Headache
CTTH: Chronic Tension Type Headache
CH: Cluster Headache
