# Supplementary material for: Osmophobia in primary headache patients: associated symptoms and response to preventive treatments
Source: J Headache Pain. 2021 Sep 18;22(1):109. doi: 10.1186/s10194-021-01327-2 (PMC8449918; doi:10.1186/s10194-021-01327-2)
Supplement: Supplementary file 1 — Additional file 1: Table 1S. Clinical variables in the headache groups. Table 2S. Clinical variables in the headache groups. Table 3S. Results of repeated measures ANOVA evaluating main effect of preventive drugs on migraine features. Table 4S. Results of repeated measures ANOVA evaluating main effect of preventive drugs on migraine features. [file 10194_2021_1327_MOESM1_ESM.docx]

| **age (years** | | | duration (years) | | headache frequency | | MIDAS |  | allodynia |  | VAS |  |
| --- | --- | --- | --- | --- | --- | --- | --- | --- | --- | --- | --- | --- |
|  | mean | ds | mean | ds | mean | ds | mean | ds | mean | ds | mean | ds |
|  |  |  |  |  |  |  |  |  |  |  |  |  |
| others | 42.61 | 3.69 | 12.63 | 3.61 | 8.48 | 2.26 | 20.87 | 9.37 | 2.17 | 0.69 | 8.48 | 0.51 |
| CH | 40,7 | 3.24 | 8,214^a^ | 3.17 | 8,92 | 1.99 | 41,35 | 8.23 | 2,92 | 0.61 | 8,78 | 0.45 |
| CM | 41.96 | 0.88 | 17.23 | 0.86 | 18.15 | 0.54 | 48.82 | 2.23 | 4.00 | 0.16 | 8.77 | 0.12 |
| CTTH | 40.22 | 2.62 | 11.91 | 2.56 | 17,64 | 1.61 | 46.86 | 6.64 | 3.17 | 0.49 | 7.52 | 0.36 |
| ETTH | 36,56 | 2.21 | 9,10 | 2.17 | 3,26 | 1.36 | 11,16 | 5.62 | 1,06 | 0.42 | 6,93 | 0.30 |
| MA | 34.02 | 2.65 | 9.50 | 2.60 | 3.56 | 1.63 | 10.78 | 6.74 | 3.08 | 0.50 | 8.39 | 0.37 |
| MO/MA | 37.49 | 1.43 | 17.89 | 1.40 | 7.00 | 0.88 | 20.10 | 3.63 | 3.49 | 0.27 | 8.67 | 0.20 |
| MO | 38.64 | 0.64 | 15.48 | 0.63 | 5.72 | 0.40 | 14.77 | 1.64 | 3.24 | 0.12 | 8.51 | 0.09 |
| Bonferroni | MA vs others p<0.05 | |  |  | CM AND CTH vs other groups p<0.01 | | CM and CTH vs MO,MA,ETH, MO/MA p<0.01 | | CM vs others, ETH,CTH,MO p<0.01 | | CH and CM vs CTH and ETH p<0.05 | |

Table 1 S Clinical variables in the headache groups

Results of Bonferroni test are showed CH: Cluster Headache; CM: Chronic Migraine; CTTH: Chronic Tension Type headache; ETTH; Episodic Tension Type Headache. MA: Migraine with Aura; MO: Migraine WithOut Aura

|  | SAS |  | SDS |  |
| --- | --- | --- | --- | --- |
|  | mean | ds | mean | ds |
|  |  |  |  |  |
| Others | 38.02 | 2.16 | 42.30 | 2.11 |
| CH | 36,64 | 1.89 | 37,00 | 1.85 |
| CM | 42.83 | 0.51 | 43.37 | 0.50 |
| CTH | 42.17 | 1.53 | 42.55 | 1.50 |
| ETH | 35,66 | 1.29 | 39,30 | 1.27 |
| MA | 37.62 | 1.55 | 39.65 | 1.52 |
| MO/MA | 39.56 | 0.84 | 41.71 | 0.82 |
| MO | 37.27 | 0.38 | 40.32 | 0.37 |
| Bonferroni | CM vs MO p<0.001 vs other groups p<0.01 | |  |  |

Table 2 S

Clinical variables in the headache groups

Results of Bonferroni test are showed

SAS and SDS: Anxiety and Depression Scales by Zung

CH: Cluster Headache; CM: Chronic Migraine; CTH: Chronic Tension Type headache; ETH; Episodic Tension Type Headache. MA: Migraine with Aura; MO: Migraine WithOut Aura

| **Headache Frequency** | | | |  |  | MIDAS |  |  |  | Pain intensity |  |  |  |
| --- | --- | --- | --- | --- | --- | --- | --- | --- | --- | --- | --- | --- | --- |
|  | N | T0 mean | SD | T1 mean | SD | T0 mean | DS | T1 mean | DS | T0 mean | DS | T1 mean | DS |
| propranol/atenolol | 33 | 9.21 | 6.24 | 5.50 | 4.64 | 27,04 | 34,378 | 10,82 | 13,425 | 8.53 | 1.08 | 8.03 | 1.38 |
| flunarizine | 120 | 9.38 | 5.85 | 5.95 | 5.11 | 22,90 | 24,810 | 13,30 | 21,055 | 8.70 | 1.13 | 8.14 | 1.50 |
| amitriptiline | 322 | 14.50 | 9.11 | 10.04 | 8.37 | 28,80 | 36,490 | 20,58 | 28,562 | 8.78 | 1.22 | 8.35 | 1.44 |
| Magnesium | 43 | 6.17 | 3.41 | 4.37 | 4.09 | 13,35 | 27,183 | 8,68 | 18,430 | 8.10 | 1.23 | 7.49 | 1.67 |
| topiramate | 167 | 15.78 | 9.38 | 11.02 | 8.70 | 33,97 | 42,668 | 22,05 | 30,191 | 8.79 | 1.13 | 8.23 | 1.47 |
| candesartan | 26 | 13.10 | 9.90 | 11.10 | 9.32 | 23,50 | 22,234 | 17,64 | 18,597 | 8.92 | 1.19 | 8.52 | 1.50 |
| Manova |  |  |  |  |  |  |  |  |  |  |  |  |  |
| main effect F |  | 108.00 |  |  |  | 41,76 |  |  |  | 64.86 |  |  |  |
| P |  | p <0.0001 |  |  |  | p<0.0001 |  |  |  | p<0.0001 |  |  |  |
| treatments F |  | 2.99 |  |  |  | 1,25 |  |  |  | 0.47 |  |  |  |
| P |  | 0.01 |  |  |  | 0,28 |  |  |  | 0.79 |  |  |  |
| Bonferroni |  | Mag vs flun, am and top p<0.01 | | | |  |  |  |  |  |  |  |  |
|  |  | Flun vs mag, am and top p<0.01 | | | |  |  |  |  |  |  |  |  |

Table 3 S

Results of repeated measures ANOVA evaluating main effect of preventive drugs on migraine features

|  | allodynia |  |  |  | SAS |  |  |  | SDS |  |  |  |
| --- | --- | --- | --- | --- | --- | --- | --- | --- | --- | --- | --- | --- |
|  | T0 mean | DS | T1 mean | DS | T0 mean | DS | T1 mean | DS | T0 mean | DS | T1 mean | DS |
| propranol/atenolol | 2.54 | 1.71 | 1.93 | 1.51 | 35.14 | 8.41 | 34.79 | 7.89 | 33.36 | 8.68 | 34.43 | 8.02 |
| flunarizine | 2.09 | 1.60 | 1.57 | 1.52 | 32.22 | 7.02 | 30.77 | 5.99 | 31.09 | 7.11 | 30.53 | 6.67 |
| amitriptiline | 2.62 | 1.71 | 2.33 | 1.70 | 38.10 | 8.47 | 36.40 | 8.07 | 36.64 | 9.52 | 35.73 | 8.50 |
| Magnesium | 1.22 | 1.18 | 0.92 | 1.12 | 32.76 | 7.17 | 31.22 | 7.64 | 30.19 | 7.50 | 29.86 | 7.41 |
| topiramate | 2.64 | 1.69 | 2.25 | 1.95 | 36.89 | 10.13 | 36.28 | 9.65 | 35.82 | 10.73 | 36.01 | 9.97 |
| candesartan | 2.45 | 1.97 | 2.00 | 1.88 | 35.95 | 8.49 | 35.77 | 8.97 | 35.73 | 8.74 | 32.45 | 7.38 |
| Manova |  |  |  |  |  |  |  |  |  |  |  |  |
| main effect F | 33.87 |  |  |  | 12,79 |  |  |  | 4,07 |  |  |  |
| P | p<0.0001 |  |  |  | p<0.0001 |  |  |  | p 0.044 |  |  |  |
| treatments F | 0.81 |  |  |  | 1,65 |  |  |  | 2,60 |  |  |  |
| P | 0.53 |  |  |  | 0,14 |  |  |  | 0,024 |  |  |  |
| Bonferroni |  |  |  |  |  |  |  |  | amitriptiline vs flunarizine and magnesium p<0.01 | | | |
|  |  |  |  |  |  |  |  |  |  |  |  |  |

Table 4 S Results of repeated measures ANOVA evaluating main effect of preventive drugs on migraine features
